# A Comparison of Urinary Mercury between Children with Autism Spectrum Disorders and Control Children

**DOI:** 10.1371/journal.pone.0029547

**Published:** 2012-02-15

**Authors:** Barry Wright, Helen Pearce, Victoria Allgar, Jeremy Miles, Clare Whitton, Irene Leon, Jenny Jardine, Nicola McCaffrey, Rob Smith, Ian Holbrook, John Lewis, David Goodall, Ben Alderson-Day

**Affiliations:** 1 North Yorkshire and York Primary Care Trust, York, United Kingdom; 2 Tees Esk and Wear Valleys NHS Foundation Trust, Durham, United Kingdom; 3 Hull York Medical School, York, United Kingdom; 4 RAND Corporation, Santa Monica, California, United States of America; 5 Food and Environment Research Agency, Sand Hutton, United Kingdom; 6 York NHS Foundation Trust, York, United Kingdom; 7 Department Of Chemistry, York University, York, United Kingdom; The University of Queensland, Australia

## Abstract

**Background:**

Urinary mercury concentrations are used in research exploring mercury exposure. Some theorists have proposed that autism is caused by mercury toxicity. We set out to test whether mercury concentrations in the urine of children with autism were significantly increased or decreased compared to controls or siblings.

**Methods:**

Blinded cohort analyses were carried out on the urine of 56 children with autism spectrum disorders (ASD) compared to their siblings (*n* = 42) and a control sample of children without ASD in mainstream (*n* = 121) and special schools (*n* = 34).

**Results:**

There were no statistically significant differences in creatinine levels, in uncorrected urinary mercury levels or in levels of mercury corrected for creatinine, whether or not the analysis is controlled for age, gender and amalgam fillings.

**Conclusions:**

This study lends no support for the hypothesis of differences in urinary mercury excretion in children with autism compared to other groups. Some of the results, however, do suggest further research in the area may be warranted to replicate this in a larger group and with clear measurement of potential confounding factors.

## Introduction

Some authors have been concerned that mercury-based preservatives in certain vaccinations [1], mercury in maternal dental fillings [2], or childhood mercury exposure from a range of environmental sources [3] may affect the brains of children, leading to autism in some individuals. This has arisen in the context of studies of neurological damage from environmental mercury or methylmercury poisoning [4], [5], and speculation as to the effect of ethylmercury in thimerosal-based vaccines [6]. Hypotheses such as these have generated much parental anxiety [7], have been implicated in reductions in childhood vaccination rates [8] and have been subsequently associated with increases in cases of measles and mumps, with significant long term implications for individuals [9]. As such, direct and rigorous testing of such hypotheses is vital not only for understanding autism, but for wider public health reasons.

Developmental problems associated with environmental mercury exposure are well documented. In a study of 63 infants in Japan with congenital mercury poisoning a range of reported problems were observed, including significant learning disabilities, limb deformities, cerebellar ataxia [4], hypersalivation, chorea and microcephaly [10]. Cerebral pathological changes showed demyelination of the pyramidal tracts, hypoplasia of the corpus callosum, widespread disturbance of brain growth and neuronal migration, neuronal and generalised cortical atrophy and underdevelopment of the granula layer of the cerebellum [5]. A further study in the Philippines where mercury is used in the gold mining industry found that prenatal exposure to mercury led to increased rates of global developmental delay [11].

It is well established that environmental mercury in high doses is very toxic [12], [13]. In Iraq in the 1970s, over 450 people died and over 5000 suffered poisoning following the use of methylmercury fungicide to treat grain [14], [15] and in Japan, industrial waste containing mercury, poisoned over 2000 people [4]. There have been several reviews examining mercury poisoning from follow up studies in Japan, Iraq, Peru, the Philippines, the Faroe Islands and the Seychelles [12], [13], [16], [17]. but, none of these have to date presented an association specifically with autistic symptomatology.

Much lower exposure to mercury than seen in these studies occurs in most societies, with one route being the use of mercury in amalgam fillings. In a study in Portugal over 500 children aged 8–10 were randomised to receive either amalgam fillings or composite fillings [18]. The amalgam group showed higher levels of creatinine-corrected urinary mercury levels at follow-up, but no differences in a range of neurobehavioural measures between the two groups over seven years follow-up. A similar study in Boston, USA followed children up for five years and came to similar conclusions [19], where children who were randomised to receive amalgam fillings were not statistically different on psychometric testing (including IQ and memory) to those receiving composite fillings.

The fear that mercury causes autism came from speculation that the use of thimerosal in certain vaccinations may have caused rates of autism diagnoses to rise [1], [6]. This was proposed despite the fact that the mercury compound it contains, ethylmercury cannot easily pass through the blood-brain barrier, as methlymercury can and is associated with few central nervous system problems in environmental health research [20]. Although one research group has published several studies linking autism rates and mercury/thimerosal [21], [22] a number of high-quality cohort and ecological studies have found no evidence to support their claims [7], [23]. Most notably, increasing rates of autism have been observed in three countries even after thimerosal was removed from their vaccination programme [24], [25].

Despite this, some authors have continued to posit mercury-related abnormalities in the development of autism. It has been hypothesised that children with autism may have a problem excreting mercury from the body [26] following evidence of reported low concentrations of mercury in the hair of 90 children with autism compared to 45 healthy controls [27]. This hypothesis exists despite the fact that other studies using hair mineral analysis have found no differences in mercury concentrations [28]–[30] and one study found increased concentrations in hair, along with other potentially hazardous metals [31].

Another excretion route is urine; if mercury excretion were impaired in ASD children, this should also be evident in their urinary levels of mercury. Whilst urine studies exploring foetal exposure in mothers who eat large amounts of mercury in fish have found no neurocognitive risk to the children of mothers eating on average 12 fish meals per week [32],[33], higher maternal mercury consumption (eating regular amounts of whale meat), has been associated with a range of subtle neurodevelopmental effects in language, attention and memory in their infants [34].

Studies that have looked specifically at the urine of children with autism have produced mixed results. Bradstreet and colleagues [35] reported higher levels of mercury in the urine of 221 children with ASD compared to controls following oral chelation treatment (to bind mercury and force excretion), which they argued was consistent with Holmes and colleagues' [27] suggestions of poor excretion and subsequent build-up of mercury levels in children with autism. Another study by the same group [36] reported increased urinary porphyrin levels related to heavy metal body-burden in a sample of ASD children. In contrast, a study by Soden and colleagues [37] found no evidence of increased levels of urinary mercury or any other heavy metals in ASD participants and controls following chelation.

None of the above studies directly measured urinary levels of mercury in children who had no form of chelation treatment. Furthermore, the Geier/Bradstreet group has only used very small control samples (*n* = 18, 14 & 5 respectively), perhaps because of the use of chelation, which is potentially harmful for children [38]. As such, there is a lack of high-quality evidence on ordinary urinary mercury levels in ASD children, at a time - with parental concerns about mercury persisting [39] - when clear and unbiased data on the issue is needed.

We set out to do a blinded study to compare urine concentrations of mercury between groups of children with autism spectrum disorders and controls. Unlike other studies of urinary mercury in autism we examined ordinary, non-treatment levels of mercury and compared them to three different control groups; mainstream schoolchildren (*n* = 115), children from special schools (*n* = 28) and ASD siblings (*n* = 40). To account for differing levels of mercury exposure across groups, the number of current amalgam fillings was recorded. Urine concentration was controlled for by correcting for creatinine. Given some authors views that heavy metals more broadly might be implicated or face excretion difficulties [36], [40], we set out to do some pilot work in this area by doing preliminary analysis on other easily measurable heavy metals in the urine. We did this in part to check whether any hypothesised problems were specific or occurred across a group of heavy metals.

## Methods

Samples of urine were collected from children with autism spectrum disorders (ASDs), ASD siblings, mainstream control children and special school control children. A special school mainly educates children with learning disabilities in the first percentile range. The sample were subsequently frozen at −80°C. Diagnoses of Childhood Autism, Atypical Autism or Asperger Syndrome were made using Research Diagnostic Criteria (RDC) from the World Health Organisation International Classification of Diseases system version 10 [41] through a multidisciplinary panel that considers all local ASD assessments. A local Autism Spectrum Disorders Forum is a mutlidsciplinary group that assesses and diagnoses all local children. All families on their database were sent information about the research from their clinician and those giving informed consent were recruited into the study. The protocol requires the RDC to be established across home, school and clinic. Where there was uncertainty the Autism Diagnostic Inventory-Revised (ADI-R) [42] and the Autism Diagnostic Observation Schedule-Generic (ADOS-G) [43] were used. Both are instruments that enhance and support ICD-10 diagnosis. For controls, healthy children without autism (or any previous assessments for ASD) from both special schools and mainstream schools were recruited in cohorts from schools supportive of the research.

Ethical and governance approval was granted by local health ethics and R&D Committees in York. Exclusion criteria included any known metabolic or neurological disorder. Children were excluded from the control group if they had received an assessment for an autism spectrum disorder or where concerns had been raised by the teacher that the child might be on the autism spectrum. Where capacity to provide written consent was reduced then those with parental responsibility provided written consent on their child's behalf with the child or young person also giving their assent.

Morning samples were collected in sterile plastic pots, sealed and then placed in a sealed plastic wrapper until collection. No sulfamic acid or surfactant was used. The urine samples were blinded with code numbers and delivered via one of several points of collection to a local Department of Clinical Biochemistry within 24 hours. Creatinine concentrations were determined and aliquots were separated and frozen at −20°C. Analysis of urine was calculated at the Food and Environment Agency at Sand Hutton and the Health and Safety Laboratory in Sheffield, as described below.

### Technical methodology

The analytical laboratories remained blinded to identity or diagnosis throughout.

#### Central Science Laboratory (Food and Environment Research Agency - Fer)

Fer's role in the project was to provide multi-element data. A random subsample (mainstream n = 24, ASD n = 11, special school n = 9) was selected by a laboratory technician blinded to diagnosis or any identifying information using randomised numbers. These urines were additionally tested for other heavy metals. Fer performed an initial screen of 25 elements which, after QA/QC assessment, was reduced to 10, i.e., lithium, vanadium, manganese, cobalt, copper, cadmium, antimony, barium, mercury and lead. Using this, the Limits of Distinction (LOD) were calculated and were all satisfactory except the LOD achievable for mercury, which was 0.9 µg/l, and so all samples were then sent to the Health and Safety Laboratory (HSL) for analysis of this specific element given lower available LODs. The Limit of Detection (LOD) describes the lowest level a target substance can be reliably detected.

Measurement of the other nine elements was by Inductively Coupled Plasma Mass Spectrometry*. All aspects of the method were performed to UKAS acceptable criteria. Fer is a regular successful participant in the relevant Series of FAPAS (proficiency testing scheme).

#### Health and Safety Laboratory Method for mercury analysis

Mercury was determined in urine samples using Inductively Coupled Plasma Mass Spectrometry (ICP-MS) Thermo Fisher Scientific X7, Series 1 (Hemel Hempstead, UK). The urine samples were defrosted at room temperature and rolled to mix. The urine samples were then diluted 1 in 10 (very small samples were diluted 1 in 5) with 5% nitric acid solution, containing 10 µg/L platinum as an internal standard and 1 mg/L gold as a stabiliser for the mercury. External quality control (EQC) samples (Bio-Rad Level 1 Lypocheck Urine) and a 5 µg/L calibration standard check were analysed at the start and end of analysis and after every ten samples. The aqueous calibration standards were in the range 0–100 µg/L. Any sample outside the calibration range would be repeated. The ICP-MS was operated in normal mode with direct nebulisation and a dedicated mercury rinse solution was aspirated containing 1 mg/L gold and 5% nitric acid.

The results for the EQC for 53 samples was 40.5±4.6 µg/L (expected range for BR 69091 38–58 µg/L). The stability of the 5 µg/L check standard was 5±0.5 µg/L. The method is UKAS accredited and acquires successful participation in both the UK TEQAS and German G-EQUAS quality assurance schemes.

In this study the LOD for mercury using a volume of 1 millilitre was 0.35 nmol/L (0.07 µg/l). The LODs for elements other than mercury were all well below the levels being found.

Analysis was performed to see if there was any relationship between mercury levels and diagnostic group. This test was performed twice: i) without creatinine correction and ii) with creatinine correction. Creatinine ratios were calculated to correct for variations in urinary mercury caused by body mass and urine concentration.

Some levels of mercury (28) in urine were below the limit of detection (LOD). In order not to lose valuable information in the analysis, we have analysed the data treating values below the LOD in two ways, firstly treating it as zero and secondly using the mercury LOD threshold value (0.35 nmol/L). This covers the spread of possible values below the limit of detection (i.e. the amount could be anything (from zero to 0.35 nmol/L). The statistical analysis therefore covers this range of possible options. In addition, data about the number of current in situ amalgam fillings was collected systematically from all participants' dental records.

### Statistical analysis

The power calculation was based on the study by Holmes and colleagues [27], who found that mercury levels (reported as parts per million) in first baby haircut was 0.47 (±0.28) for Autistic and 3.63 (±3.56) for controls. To detect a difference of 3 between the Autistic group in this study and the control groups, 33 patients are required per group, based on 80% power and 5% significance.

The four groups were compared using Kruskall Wallis tests for continuous data and Chi-Square for categorical data. A p-value of <0.05 was considered to indicate statistical significance. To adjust of the effects of age, gender and number of fillings multiple regression was undertaken. All analyses were performed on SPSS (version 18). Kruskal-Wallis tests were utilised instead of parametric equivalents due to skew in the raw data.

## Results

Included in the study were 251 children: 54 children on the autism spectrum, 155 children without autism (121 were currently attending mainstream school and 34 children were attending a special school), and 42 children who were the siblings of those with ASD.

There was a significant difference between the groups for age (F(3) = 20.726, p<0.001), and gender (Chi(3) = 15.900, p = 0.001). The number of fillings was slightly lower in ASD children (Table 1), although this was not statistically significant (*KW* (3) = 3.907, *p* = 0.272), even after grouping participants into those with or without fillings (Chi(3) = 3.893, p = 0.273).

**Table 1:** Urinary mercury, creatinine and numbers of amalgam dental fillings.

	ASD	Special School	Mainstream School	Sibling
**Urinary mercury levels**				
N	54	34	121	42
Age (*M/SD*)	9.6 (3.6)	12.6 (3.5)	8.8 (2.4)	12.1 (3.6)
Sex (N/% Male)	42/53 (79%)	18/34 (53%)	65/119 (55%)	17/42 (41%)
Returned urines from consented children	47 (87%)	28 (82%)	115 (95%)	40 (95%)
Number below LOD of 0.35	2 (4%)	3 (11%)	15 (13%)	8 (20%)
**Number of amalgam dental fillings N**	41	28	98	29
No fillings	36 (88%)	22 (79%)	72 (74%)	24 (83%)
1 or more	5 (12%)	6 (21%)	26 (26%)	5 (17%)
*M* (*SD*)	0.4 (1.3)	0.5 (1.3)	0.7 (1.4)	0.3 (0.8)
**Mercury ** ***M*** ** (** ***SD*** **) nmol/l N**	47	28	115	40
Values below LOD treated as 0.35	6.61 (6.95)	6.43 (5.80)	6.23 (7.14)	4.38 (4.34)
***M*** ** (** ***SD*** **) Median (1QR)**	5.20 (2.80, 6.90)	4.65 (2.35, 9.20)	4.00 (1.80, 7.10)	3.35 (0.50. 6.45)
Values below LOD treated as 0	6.60 (6.96)	6.39 (5.84)	6.18 (7.18)	4.30 (4.42)
***M*** ** (** ***SD*** **) Median (1QR)**	5.20 (2.80, 6.90)	4.65 (2.35, 9.20)	4.00 (1.80, 7.10)	3.35 (0.50, 6.45)
**Creatinine**	10.35 (5.27)	11.11 (5.15)	11.22 (4.62)	11.75 (4.86)
***M*** ** (** ***SD*** **) Median (1QR)**	10.05 (6.60, 14.60)	10.40 (7.50, 14.30)	10.20 (8.00, 13.30)	10.60 (8.00, 15.10)
**Mercury nmol/l/Creatinine mmol/l..N**	47	28	115	40
**Mercury nmol/l** (Values below LOD treated as 0.35)/**Creatinine mmol/l**	0.94 (1.35)	0.90 (1.48)	0.58 (0.57)	0.52 (0.62)
***M*** ** (** ***SD*** **) Median (1QR)**	0.52 (0.32, 0.93)	0.42 (0.21, 0.94)	0.38 (0.19, 0.76)	0.33 (0.04, 0.58)
**Mercury nmol/l** (Values below LOD treated as 0.35)/**Creatinine mmol/l**	0.94 (1.35)	0.89 (1.49)	0.57 (0.58)	0.52 (0.63)
***M*** ** (** ***SD*** **) Median (1QR)**	0.52 (0.32, 0.93)	0.42 (0.21, 0.94)	0.38 (0.19, 0.76)	0.33 (0.03, 0.58)

No mercury samples fell outside the calibration range.

Table 1 gives data on urine mercury level for the ASD and control groups. Mercury levels were attained for 230 of the 251 children (92%). Of these 28 results that were below LOD were replaced by either 0.35 or zero. (as described above).

There were no significant differences between the four groups in uncorrected mercury level regardless of how the values below the LODs were treated [mercury level with blanks given zero (KW(3) = 5.135, p = 0.162), or mercury level with blanks given 0.35 (KW(3) = 5.223, p = 0.156]. There was no difference between the groups and creatinine levels (KW(3) = 1.734, p = 0.630).

**Figure 1:**
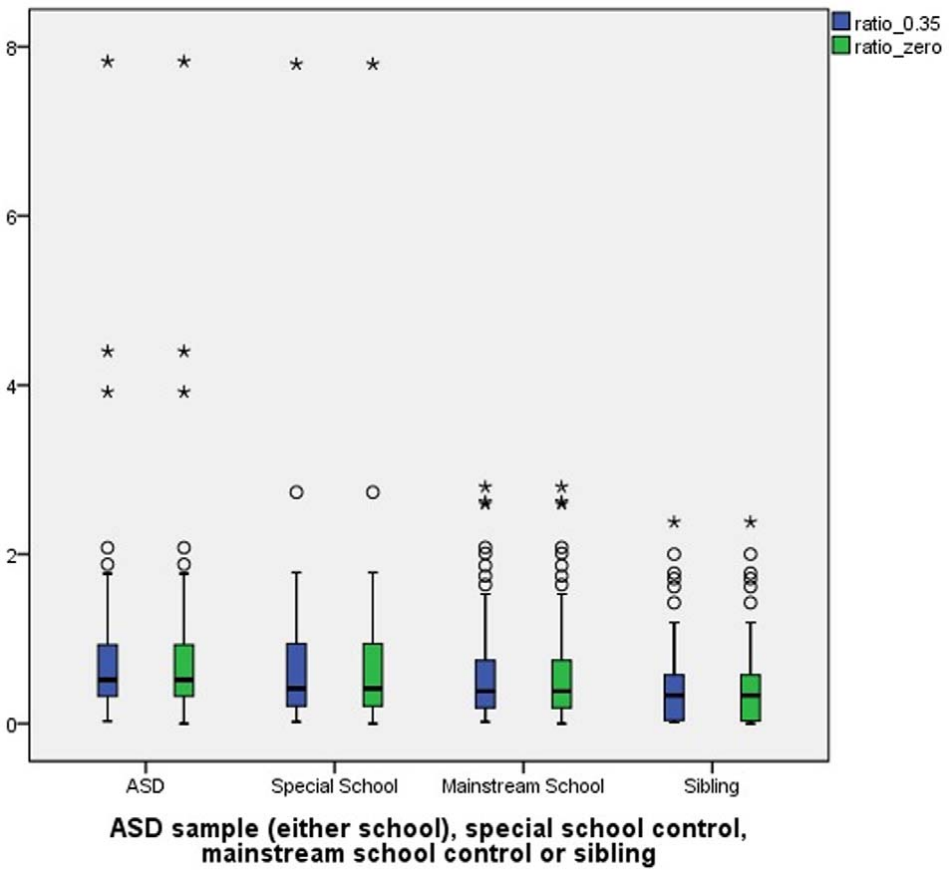
Boxplot of Mercury/Creatinine ratio. Boxes represent interquartile range (bar = median). O = 1.5–3 box lengths and * = more than 3 box lengths from the box.

Table 1 and Figure 1 show the data for the urine mercury corrected for creatinine. There were no significant differences between the four groups regardless of how the values below the LODs were treated (mercury level with blanks given zero (KW(3) = 6.889, p = 0.076) or mercury level with blanks given 0.35 (KW(3) = 7.450, p = 0.059)). Even after removing outliers with extreme values (children with values more than 3 box lengths from the upper or lower edge of the box. (The box length is the interquartile range) there were no significant differences between groups (mercury level with blanks given zero (KW(3) = 5.738, p = 0.125) or mercury level with blanks given 0.35 (KW(3) = 6.333, p = 0.096)).

After adjusting for age, gender and number of fillings, there was still no statistically significant difference between the groups (mercury level with blanks given zero: F (3) = 2.587, p = 0.056; mercury level with blanks given 0.35: F (3) = 2.570, p = 0.056), even after removing extreme values (mercury level with blanks given zero: F (3) = 0.897, p = 0.444; mercury level with blanks given 0.35: F (3) = 0.867, p = 0.459).

Tests of other heavy metals find no differences between groups. This includes lithium (p = 0.344), vanadium (p = 0.951), manganese (p = 0.613),cobalt (p = 0.392, copper (p = 0.391), cadmium (p = 0.586), antinomy (p = 0.216), barium (p = 0.328) and lead (p = 0.203)*.

## Discussion

The hypothesis that mercury poisoning (either through increased exposure or reduced excretion) may cause autism is a cause of anxiety to many parents. It may prompt them to change vaccination behaviour and in some rare cases to use oral chelating agents, both of which pose a risk to the child [38].

The widespread but much lower dose exposure of children to ethylmercury in some vaccinations containing thimerosal has been studied epidemiologically [44] and to date has not been shown to be associated with autism [45]. Blood levels of mercury after vaccination also appear to be very low [46]. Most tellingly, rates of diagnosis for autism continued to rise, after thimerosal use in paediatric vaccines in the developed world was discontinued in 2001 [47].

We found no statistically significant differences in urinary mercury corrected for creatinine, between the groups compared to the control groups, mainstream children and siblings. The other heavy metals showed no differences that would have encouraged us to test the whole group for these elements, or to suggest broader heavy metal metabolism problems.

We interpret our findings with caution. The results appear to be influenced strongly by a small number of extreme values in the ASD and special school group but not the other two groups (see Figure 1). There is no significant difference between groups when the extreme values are removed The ASD or LD groups do not appear to have more or less amalgam fillings, although it is known that amalgam fillings affect urinary mercury content. A lower creatinine in the ASD group (not statistically significant) raises the mercury to creatinine ratio in this subgroup, but not to statistically significant levels. Since creatinine is dependent on age, race, body mass index, fat free mass [48] and glutathione metabolism/genetics [49] future studies should include measures of these variables for analysis.

This study makes no comparisons of brain mercury levels between the two groups. The finding by Holmes and colleagues [27] that mercury levels in the hair were lower are difficult to reconcile with our results but it could be that their ASD children had lower exposure to amalgam fillings or (possible though unlikely) impaired selective hair excretion in the absence of impaired urinary excretion.

The present study has limitations that must be considered. The sample size is relatively small. Given the KW and P scores there is a possibility that there is a type II error with inadequate power. Our data can be used to calculate numbers needed for a future adequately powered study. Whilst collection of 24-hour urines may have improved accuracy, such samples are very difficult to collect in children with learning disability and autism and research shows good correlations between spot urines and 24-hour collections [50].There was variability of urine collection rates between participant groups. The sibling group and mainstream children provided higher sample rates than those for special school children and ASD children. These groups had slightly lower rates mainly because of communication problems and difficulty obtaining co-operation. However, overall sample rates were good for a study of this kind, and a notable strength of the study is its large sample of controls in comparison to previous urinary studies [35], [36]. This field needs further high quality, large sample work that collects good data on age, diet, BMI and amalgam fillings.

This study does not lend support for widespread mercury metabolism problems in autism, but given small numbers of outliers it does suggest further research is warranted to better understand whether a subgroup with autism or learning disabilities have mercury poisoning or excretion difficulties. Recent research has however found no association between autism and genes polymorphisms responsible for controlling the transport and response to body mercury [51]. Given that research shows that environmental mercury is toxic to humans, avoiding mercury exposure remains intuitively sensible. However, a clear causal link to autistic spectrum disorders remains unlikely.

*Further details are available from the corresponding author.
